# Gender-differences in the associations between circulating creatine kinase, blood pressure, body mass and non-alcoholic fatty liver disease in asymptomatic asians

**DOI:** 10.1371/journal.pone.0179898

**Published:** 2017-06-30

**Authors:** Chih-Hsuan Yen, Kuang-Te Wang, Ping-Ying Lee, Chuan-Chuan Liu, Ya-Ching Hsieh, Jen-Yuan Kuo, Bernard E. Bulwer, Chung-Lieh Hung, Shun-Chuan Chang, Shou-Chuan Shih, Kuang-Chun Hu, Hung-I Yeh, Carolyn S. P. Lam

**Affiliations:** 1 Division of Cardiology, Department of Internal Medicine, Mackay Memorial Hospital, Taipei, Taiwan; 2 Mackay Medicine, Nursing and Management College and Taipei Medical University, Taipei, Taiwan; 3 Division of Cardiology, Department of Internal Medicine, Mackay Memorial Hospital, Taitung branch, Taiwan; 4 Mackay Medical College, New Taipei County, Taiwan; 5 Graduate Institute of Health Care Organization Administration, College of Public Health National Taiwan University, Taipei, Taiwan; 6 Health Evaluation Center, Mackay Memorial Hospital, Taipei, Taiwan; 7 Department of Medical Technology, Yuanpei University of Science and Technology, Hsin-Chu, Taiwan; 8 Department of Anesthesiology, Peking University First Hospital, Beijing, China; 9 Nonivasive Cardiology, Brigham and Women’s Hospital, Boston, Massachusetts, United States of America; 10 Massachusetts College of Pharmacy and Health Sciences, Boston, Massachusetts, United States of America; 11 Division of Gastroenterology, Department of Internal Medicine, MacKay Memorial Hospital, Taipei, Taiwan; 12 National Heart Centre Singapore and Duke-National University of Singapore Medical School, Singapore, Singapore; Universite du Quebec a Montreal, CANADA

## Abstract

**Background:**

Creatine kinase (CK) is a pivotal regulatory enzyme in energy metabolism linked to both blood pressure and cardio-metabolic components. However, data is lacking in a large population of asymptomatic Asians.

**Methods and results:**

Cardio-metabolic assessment including anthropometric measures and non-alcoholic fatty liver disease (NAFLD) were evaluated by abdominal echo in 4,562 consecutive subjects who underwent an annual health survey. Serum CK levels were related to blood pressure components [systolic blood pressure (SBP), diastolic blood pressure (DBP), and pulse pressure (PP)], anthropometric measures, and excessive adiposity in liver as indicated by NAFLD. Circulating CK levels ranged from 4 to 1842 IU/L (mean [SE]: 108.7 [1.1] IU/L) in the study population which consisted of 2522 males (mean age: 48.7 ± 11.2) and 2040 females (mean age: 49.4±11.5). In general, male subjects presented with higher circulating CK levels than females (mean ± SE: 127.3 ± 1.5 vs. 85.5 ± 1.3 IU/L, respectively, p < .001). Gender-differences in circulating CK levels were also observed with increasing age, which showed a more pronounced positive relationship with age in female subjects (gender interaction: p < .05). Furthermore, an elevated circulating CK level was independently associated with higher blood pressure, waist circumference and fat mass (FM), greater body mass index (BMI), increased lower estimated glomerular filtration rate (eGFR) and presence of NAFLD in multivariate analysis (all p < .05), with CK elevation more pronounced with greater BMI and FM in males compared with females (sex interaction: p < .05).

**Conclusion:**

In a large asymptomatic Asian population, circulating CK levels were increased with more advanced age, higher blood pressure, and greater body mass with gender differences. Our findings may be useful in interpreting elevated CK from subjects free of ongoing myocardial damage.

## Introduction

Creatine kinase (CK) is a central regulatory enzyme in energy metabolism, catalyzing the transfer of the phosphoryl group between creatine and adenosine diphosphate [1]. In highly metabolic tissues, CK rapidly restores adenosine triphosphate to provide energy for processes such as skeletal, cardiac, and vascular smooth muscle contraction. Circulating CK levels have been shown to be independently associated with blood pressure in the general population [2], suggesting that high CK activity may be related to enhanced energy metabolism in vascular smooth muscle and greater vasopressor effects.

Ethnic differences in circulating CK, with higher levels in blacks compared with white populations, may explain the elevated risk of hypertension in the former [3,4]. This has not been systemically examined in large Asian populations. Increased CK activity has also been linked to abnormal skeletal muscle metabolism in overweight and obese women [5]. Since skeletal muscle is known to play a key role in glucose and lipid homeostasis, significant metabolic dysfunction in obese and inactive individuals has been implicated in the development of glucose intolerance, dyslipidemia, diabetes mellitus, and metabolic syndrome [6–8].

Owing to the fact that routine measure of body mass index (BMI) or weight may not accurately reflect increased adiposity (total body fat burden) or visceral adipose tissue amount in any given overweight or obesity individual, body fat composition had been proposed as a relatively reliable estimate in these components [9]. Further, accumulated hepatic fat in the absence of excessive alcohol use as non-alcoholic fatty liver disease (NAFLD) with nearly 10% prevalence from population-based Asian cohort had been tightly linked to metabolic derangements and may provide an alternative adiposity measure [10–12].

The underlying hypothesis of this study was that higher circulating levels of CK at rest would be associated with higher blood pressure, greater adiposity or visceral obesity, and biochemical evidence of metabolic disturbance (raised blood sugars and lipids) even among asymptomatic individuals. This study also aimed to investigate whether gender differences accompanied these associations [5] in a large sample of asymptomatic Asian adults.

## Methods

### Study population

This study was approved by local ethical institutional committee (Mackay Memorial Hospital) for retrospective data analysis without informed consent of study participants (IRB No: 09MMHIS037). This cross-sectional observational retrospective analysis consisted of 4,562 consecutive subjects (mean age 49 ± 11.3, 44.7% female) participating in an annual health survey conducted at a health evaluation center at a tertiary medical center located in Taipei, Taiwan between January 2005 and December 2008. Data security was guaranteed and all authors had no access to patient identifying information before and after data analysis. Study participants involved in this study were not under clinical service of current study physicians or researchers.

Detailed medical histories using structured questionnaires, physical examination, and chest radiography were performed in all participants. Baseline characteristics and related anthropometric indices, including age, height, weight, and BMI were obtained, as well as fasting blood sugar, hepatic and renal function tests, lipid profiles, CK levels, and complete blood cell counts after overnight fasting status for 8–12 hours. CK level was examined in relation to several key baseline measurements, including age, various blood pressure components [systolic blood pressure (SBP), diastolic blood pressure (DBP), and pulse pressure (PP)], body size as determined by body mass index (BMI), clinical measures of excessive visceral fat deposits, and gender differences.

Patients with a history of coronary artery bypass surgery, rheumatic heart disease, overt heart failure symptoms, atrial fibrillation, previous pacemaker implantation, and overt renal insufficiency (creatinine > 2.5 mg/dl) were excluded. Diabetes mellitus (DM) was defined as HbA1c >6.5 for more than 2 occasions, known history of DM, or any current usage of DM medication. Hyperlipidemia treatment was defined as known history of any anti-lipid medication usage (e.g. statin). Renal function was expressed in terms of estimated glomerular filtration rate [eGFR]. Patients who had hypertension were defined as those who were on anti-hypertensive agents or had a SBP ≥ 140 mmHg and/or DBP ≥ 90 mmHg.

### Measurement of serum CK concentrations

Blood samples for serum analyses were collected by nurses blinded to the study protocol and all samples were kept in serum-separator tubes at room temperature with barrier gel and EDTA K2 as an anticoagulant. All subjects were asked to avoid vigorous exercise or intramuscular injection 48 hours prior to study enrollment. The CK samples were consecutively analyzed in an automated clinical chemistry analyzer (Modular P, Roche). Sample analyses were performed within 6 hours of withdrawal and analysis was performed by photometry using an enzymatic method (CK-NAC; Roche Diagnostics, Mannheim, Germany). The analytical coefficient of variation (COV) was 1.4%. All the procedures were performed at the Department of Health Center, Mackay Memorial Hospital.

#### Determination of body anthropometric variables and non-alcoholic fatty liver disease (NAFLD)

Total body fat percentage was determined using a foot-to-foot bioelectrical impedance method (Tanita-305 Body-fat Analyzer, Tanita Corp, Tokyo, Japan) with technicians blinded to baseline demographics. This technique is based on the principle that tissues conduct electricity based on their water and electrolyte content. For example, fat and bone tissues are relatively nonconductive compared to muscle tissue. Recognizing that higher selective deposition of visceral adipose tissue, rather than subcutaneous adipose tissue, is associated with increased cardiometabolic risk [9], waist circumference was used as an index of visceral adiposity in current work.

Excessive adiposity in liver tissue (defined as hepatic adiposity) by abdominal sonography assessment was performed using a Toshiba Nemio SSA-550A instrument (Toshiba, Tochigi-ken, Japan) by hepatologists blinded to study protocols. Briefly, liver pathology was graded semi-quantitatively as absent, mild, moderate, or severe according to the echogenicity of the hepatic parenchyma [10]. As there may be significant subjectivity in the diagnosis and assessment of fatty liver by abdominal ultrasound, hepatic adiposity in the context of significant non-alcoholic fatty liver disease (NAFLD) was further defined as at least moderate-to-severe degree fatty liver disease without regular alcohol intake (defined as ≧1 regular drink per week).

### Statistical analysis

Baseline characteristics of the entire population were analyzed as quartiles of CK. Continuous data [expressed as mean ± standard deviation (SD)] were analyzed using a *t*-test or Cuzick's nonparametric trend test across ordered CK-stratified quartiles groups, with categorical data were analyzed using a chi-square test or Fisher’s exact test, as appropriate (results expressed as ratios). Baseline demographics [including age, gender, blood pressure, various anthropometric measures (BMI, waist circumference, body fat mass), NAFLD status, biochemical data (such as fasting sugar, lipid profiles), renal function (in terms of eGFR), medical histories and lifestyle behaviors] were all examined using univariate models to identify the independent associations between these clinical covariates and serum CK level, with CK as dependent variable. Owing to the relatively large variations in circulating CK levels, median values with inter-quartile ranges as well as standard errors [SEs] were also provided. All clinical covariates with statistical significance based on univariate models (p < .05) were then entered into multivariate models, and gender interactions were further explored when determining circulating CK levels. Non-parametric LOWESS smoothing methods were computed to express the unknown curve regression estimates between circulating CK levels and age, BMI, waist and various adiposity measures. Owing to the collinearity of various body size or adiposity measures (BMI, waist circumference, body fat mass, and NAFLD), these variables were included separately in multivariate models. For the same reasons, one blood pressure component (SBP, DBP and PP entered sequentially) were examined in each multivariate model. All *p* values were two-sided, with values less than .05 considered statistically significant.

## Results

### Clinical characteristics by CK quartiles

Circulating CK levels ranged from 4 to 1842 IU/L with a mean value of 108 IU/L (SD: 68.1; SE: 1.11). Across CK quartiles (Table 1), there were no significant differences in age, but there were a greater proportion of men with increasing CK quartiles compared with women (from Q1 to Q4: 28.1 to 80.2% in men, *X*^*2*^ < 0.001). Elevated CK levels were associated with higher BMI (r = 0.19), greater body fat mass (r = 0.08), and higher blood pressure components (r = 0.15, 0.14, and 0.1 for SBP, DBP and PP, respectively; all p < .001). Higher fasting glucose and HbA1c were observed in the first quartile, with similar levels observed in the remaining three groups. Furthermore, greater circulating CK was modestly associated with lower eGFR (r = -0.17), higher prevalence of hypertension (p = .008), higher prevalence of NAFLD (p < .001) and hyperlipidemia treatment (p < .001).

**Table 1:** Baseline characteristics stratified by quartiles of CK.

	Q1(CK)	Q2(CK)	Q3(CK)	Q4(CK)	
Serum Levels (IU/L)	<69	69–93	93–128	> = 128	
Total N = 4562	N = 1182	N = 1135	N = 1126	N = 1119	Trend P value
Age (year)	48.8±12.2	48.9±11.1	49.3±11.0	49.2±11.2	p = 0.1
Male (%)	332 (28.1%)	533 (46.9%)	759 (67.4%)	898 (80.2%)	P<0.001
BMI (kg/m2)	22.9±3.3	23.4±3.3	24.2±3.2	25.1±3.5	P<0.001
SBP (mmHg)	118.6±17.2	119.5±17.0	122.2±16.9	124.2±17.0	P<0.001
DBP (mmHg)	73.1±10.3	74.0±10.2	75.5±9.9	76.6±10.4	P<0.001
Pulse Pressure (mmHg)	45.5±11.2	45.5±11.2	46.6±11.9	47.4±12.1	P<0.001
Heart rate (beat/minutes)	72.1±9.4	71.6±9.2	72.0±10.1	71.4±9.7	P = 0.1
Waist Circumference (cm)	77.4±9.9	79.7±10.1	82.3±9.6	85±9.6	P<0.001
Fat Mass, kg	16.7±6.3	16.8±6.2	17.2±6.2	18±6.6	P<0.001
Fat Free Mass, kg	41.9±7.4	45.2±8.4	48.6±8.3	51.8±8.3	P<0.001
NAFLD (%)	68 (5.8%)	79 (7%)	89 (7.9%)	122 (10.9%)	P<0.001
Fasting Sugar (mg/dL)	101.2±34.2	98.7±24.2	99.2±23.9	99.6±22.5	P<0.001
HbA1c (%)	5.69±1.3	5.63±1.0	5.62±0.8	5.6±0.8	P<0.001
HbA1c > = 9.0, %	37 (3.7%)	22 (2.3%)	12 (1.2%)	12 (1.3%)	P<0.001
Uric acids (mg/dL)	5.6±1.5	5.9±1.5	6.3±1.5	6.7±1.6	P<0.001
Triglyceride (mg/dL)	121.4±104.3	120.1±100.5	126.1±86.3	132.3±117.9	P<0.001
Cholesterol (mg/dL)	191.1±37.9	195.5±36	196.2±33.2	198.3±36.8	P<0.001
HDL-C (mg/dL)	56.3±15.1	56.3±15.6	54.2±15.1	52.7±14.2	P<0.001
LDL-C (mg/dL)	117.8±33.2	122.6±32.8	125.5±31	128±33.6	P<0.001
eGFR (mL/min/1.73 m^2^)	92±19.6	87.3±17	84.8±15.9	82.4±16.8	P<0.001
Hypertension (%)	126 (11.1%)	104 (9.4%)	135 (12.3%)	151 (13.9%)	P = 0.008
Smoking (%)	148 (12.5%)	164 (14.5%)	208 (18.5%)	210 (18.8%)	P<0.001
Diabetes Mellitus (%)	99 (8.4%)	86 (8.6%)	96 (9.3%)	92 (9.2%)	P = 0.844
Hyperlipidemia treatment (%)	18 (1.5%)	17 (1.5%)	39 (3.5%)	43 (3.8%)	P<0.001

Base characteristic data, values are mean±SD. Abbreviations: BMI = body mass index; SBP = systolic blood pressure; DBP = diastolic blood pressure; HDL-C = high density lipid-cholesterol; LDL-C = low density lipid-cholesterol; NAFLD = Non-alcoholic fatty liver disease; eGFR = estimated glomerular filtration rate; CK = creatine kinase. Q: quartile groups.

### Univariate and multivariate associations of circulating CK levels

**Fig 1:**
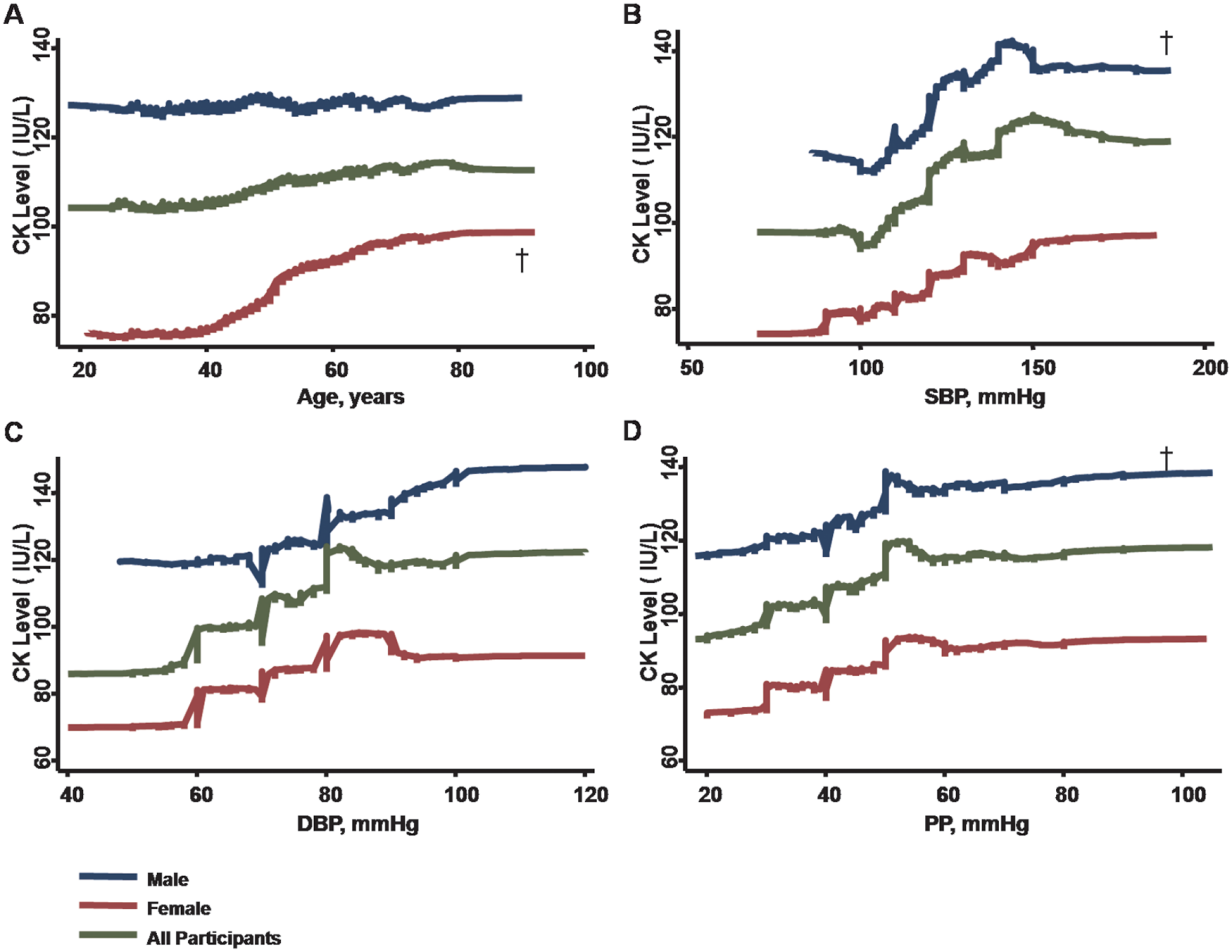
With increased age, serum CK levels decrease in men but exhibit a significantly steeper rise in the women (panel A, p <0.05 for gender interaction). Higher CK levels are also associated with increasing BP components; greater SBP and PP demonstrate steeper rise in men than in women (panel B-D). † denotes p <0.05 for gender interaction.

**Fig 2:**
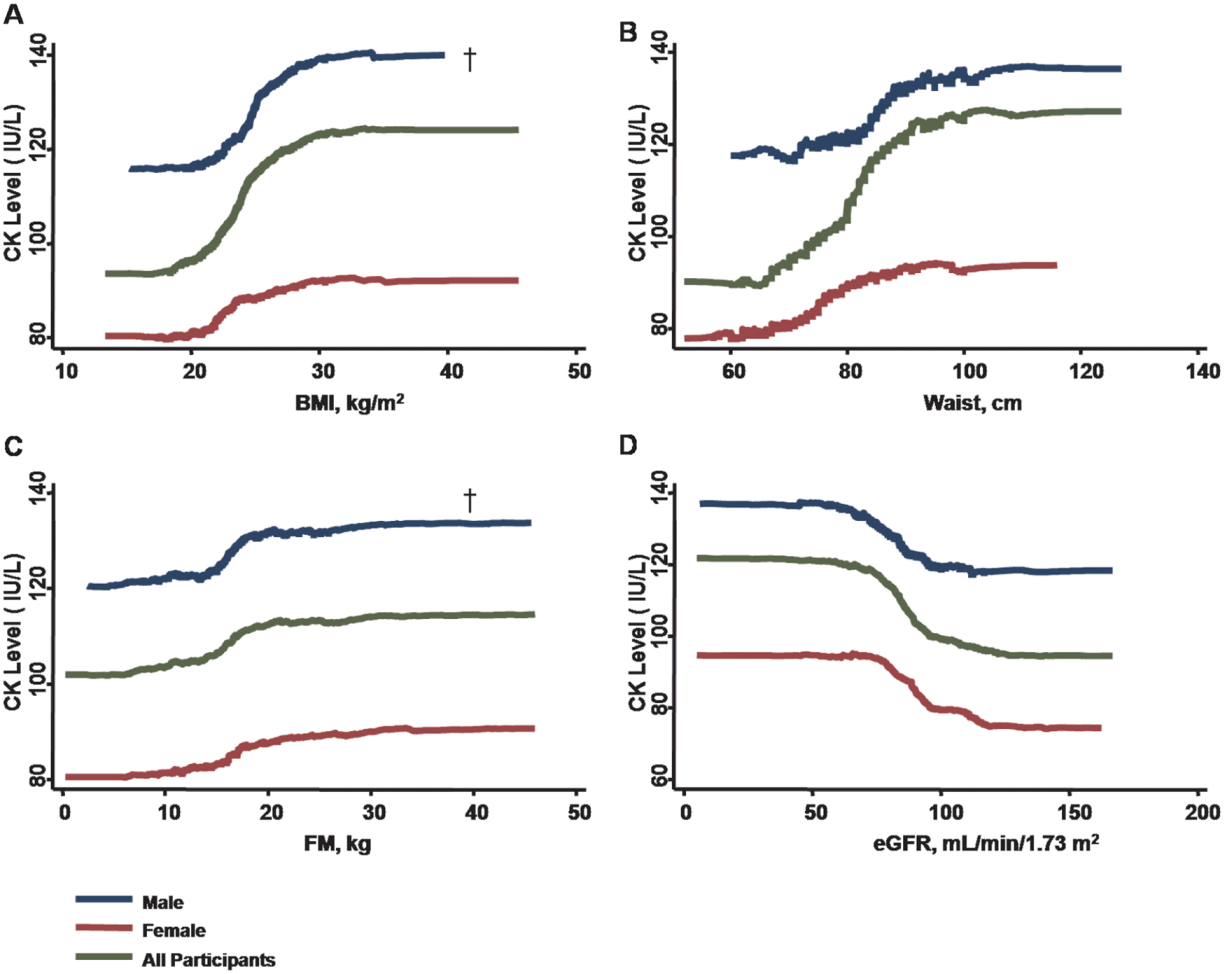
Both increased body size (in terms of greater BMI or larger waist circumference) are associated with higher serum CK levels, with more prominent rise with greater BMI in males rather than in females (panel A & B, p <0.05 for gender interaction). Higher FM levels and worsening renal function (in the context of lower eGFR) are also associated with increasing CK levels, with males exhibiting a steeper rise with higher FM compared with females (panel C, p <0.05for sex interaction). Instead, worsening eGFR does not show gender differences (panel D).

Using univariate models, increasing age, higher blood pressure components, larger anthropometric measures or index of visceral adiposity (including BMI, waist circumference, and body fat mass), and worse eGFR were all associated with higher CK levels (Table 2, **all p < .05. see also** Figs 1 and 2). Hypertension, hyperlipidemia treatment, current smoker, and presence of NAFLD were all significantly associated with higher CK levels (all p < .05) (Table 2).

**Table 2:** Univariate associations of CK with baseline characters and adiposity measures.

Predictors	*Coef*.	*95% CI*	*P value*
Age (per 10 years +)	2.68	0.77 to 4.59	0.006
Gender, Male	41.77	37.55 to 45.98	<0.001
BMI (per unit kg/m^2^+)	4.22	3.60 to 4.84	<0.001
SBP (per 10 mmHg +)	6.47	5.21 to 7.72	<0.001
DBP (per 10 mmHg +)	10.07	7.98 to 12.16	<0.001
PP (per 10 mmHg +)	6.22	4.35 to 8.09	<0.001
Heart rate (per beat/minutes +)	0.06	-0.17 to 0.28	0.625
Waist Circumference (per unit cm +)	1.70	1.49 to 1.90	<0.001
Fat Mass (per unit kg +)	0.74	0.28 to 1.27	0.008
Fasting Sugar (per unit mg/dL +)	0.01	-0.07 to 0.09	0.758
HbA1c (per unit %+)	1.39	-1.05 to 3.83	0.264
Uric acids (per unit mg/dL+)	9.43	8.09 to 10.77	<0.001
Triglyceride (per unit mg/dL+)	0.04	0.02 to 0.06	<0.001
Cholesterol (per unit mg/dL+)	0.07	0.01 to 0.13	0.017
HDL-C (per unit mg/dL+)	-0.48	-0.63 to -0.33	<0.001
LDL-C (per unit mg/dL+)	0.12	0.05 to 0.19	0.001
eGFR (per unit mL/min/1.73 m^2^ +)	-0.72	-0.84 to -0.60	<0.001
NAFLD	14.87	11.48 to 18.25	<0.001
Hypertension	20.69	15.77 to 25.61	<0.001
Smoking	9.25	3.31 to 15.19	0.002
Diabetes Mellitus	0.1	-0.07 to 0.17	0.424
Hyperlipidemia treatment	19.12	5.34 to 32.91	0.007

Abbreviations: BMI = body mass index; BP = blood pressure; FM = fat mass; eGFR = estimated glomerular filtration rate; CK = creatine kinase; SBP = systolic blood pressure; Coef = coefficient; CI = confidence interval. *Note*: Regression coefficients (*β*) represent the change in mean difference in CK (in IU/L) per 1-SD difference in each continuous predictor variable. *Other abbreviations as* Table 1.

The associations between CK levels and other biochemical data are further shown in Table 2. After adjusting for medical histories and active smoking, higher circulating CK level was consistently associated with age, male gender, higher SBP, greater anthropometric estimates or indexes of adiposity (BMI, waist circumference, body fat mass and NAFLD), and worse renal function (lower eGFR) (all p < .05) in multivariate models (Table 3). Multivariate results with blood pressure components other than SBP (i.e., DBP and PP) entered into the models are further listed in S1 Table. PP instead of DBP was also positively associated with higher circulating CK levels, even after accounting for other clinical covariates including age, gender, body adiposity measures, eGFR, medical histories, and smoking status.

**Table 3:** Multivariate associations of CK with SBP and various adiposity measures.

**Predictors**	***Coef***.	***95% CI***	***P value***
**BMI (kg/m**^**2**^**)**^#^			
Age (per 10 years +)	-2.74	-4.45 to -1.04	0.002
Gender, Male	35	31.3 to 38.7	<0.001
SBP (per 10 mmHg +)	1.68	0.3 to 3.06	0.017
BMI (per unit +)	2.61	1.88 to 3.16	<0.001
eGFR (per 10 unit +)	-3.8	-4.83 to -2.78	<0.001
**Waist (cm)**^#^			
Age (per 10 years +)	-3.29	-5.01 to -1.58	<0.001
Gender, Male	31.1	25.4 to 35.6	<0.001
SBP (per 10 mmHg +)	1.88	0.51 to 3.25	0.007
Waist (per 10 unit +)	7.44	5.48 to 9.41	<0.001
eGFR (per 10 unit +)	-3.75	-4.77 to -2.73	<0.001
**Fat Mass (kg)**^#^			
Age (per 10 years +)	-2.03	-3.75 to -0.30	0.021
Gender, Male	39.4	35.8 to 43.1	<0.001
SBP (per 10 mmHg +)	1.98	0.59 to 3.36	0.005
FM (per 5kg +)	4.56	3.21 to 5.91	<0.001
eGFR (per 10 unit +)	-3.75	-4.79 to -2.71	<0.001
**NAFLD**^#^			
Age (per 10 years +)	-2.83	-4.98 to -0.67	0.01
Gender, Male	37.2	32.7 to 41.7	<0.001
SBP (per 10 mmHg +)	3.26	1.54 to 4.98	<0.001
NAFLD	15.89	8.07 to 23.7	<0.001
eGFR (per 10 unit +)	-4.73	-6.02 to -3.44	<0.001
**Predictors**	***Coef***.	***95% CI***	***P value***
**BMI (kg/m**^**2**^**)**^#^			
Age (per 10 years +)	-2.74	-4.45 to -1.04	0.002
Gender, Male	35	31.3 to 38.7	<0.001
SBP (per 10 mmHg +)	1.68	0.3 to 3.06	0.017
BMI (per unit +)	2.61	1.88 to 3.16	<0.001
eGFR (per 10 unit +)	-3.8	-4.83 to -2.78	<0.001
**Waist (cm)**^#^			
Age (per 10 years +)	-3.29	-5.01 to -1.58	<0.001
Gender, Male	31.1	25.4 to 35.6	<0.001
SBP (per 10 mmHg +)	1.88	0.51 to 3.25	0.007
Waist (per 10 unit +)	7.44	5.48 to 9.41	<0.001
eGFR (per 10 unit +)	-3.75	-4.77 to -2.73	<0.001
**Fat Mass (kg)**^#^			
Age (per 10 years +)	-2.03	-3.75 to -0.30	0.021
Gender, Male	39.4	35.8 to 43.1	<0.001
SBP (per 10 mmHg +)	1.98	0.59 to 3.36	0.005
FM (per 5kg +)	4.56	3.21 to 5.91	<0.001
eGFR (per 10 unit +)	-3.75	-4.79 to -2.71	<0.001
**NAFLD**^#^			
Age (per 10 years +)	-2.83	-4.98 to -0.67	0.01
Gender, Male	37.2	32.7 to 41.7	<0.001
SBP (per 10 mmHg +)	3.26	1.54 to 4.98	<0.001
NAFLD	15.89	8.07 to 23.7	<0.001
eGFR (per 10 unit +)	-4.73	-6.02 to -3.44	<0.001

Abbreviations AS Tables 1 & 2. Coef = coefficient; CI = confidence interval. *Note*: Regression coefficients (*β*) represent the change in mean difference in CK (in IU/L) per 1-SD difference in each continuous predictor variable. *Other abbreviations as* Table 1.

^#^ Further adjusted for current smoker, hypertension, hyperlipidemia treatment and diabetes history.

### Sex-specific correlates of CK

**Fig 3:**
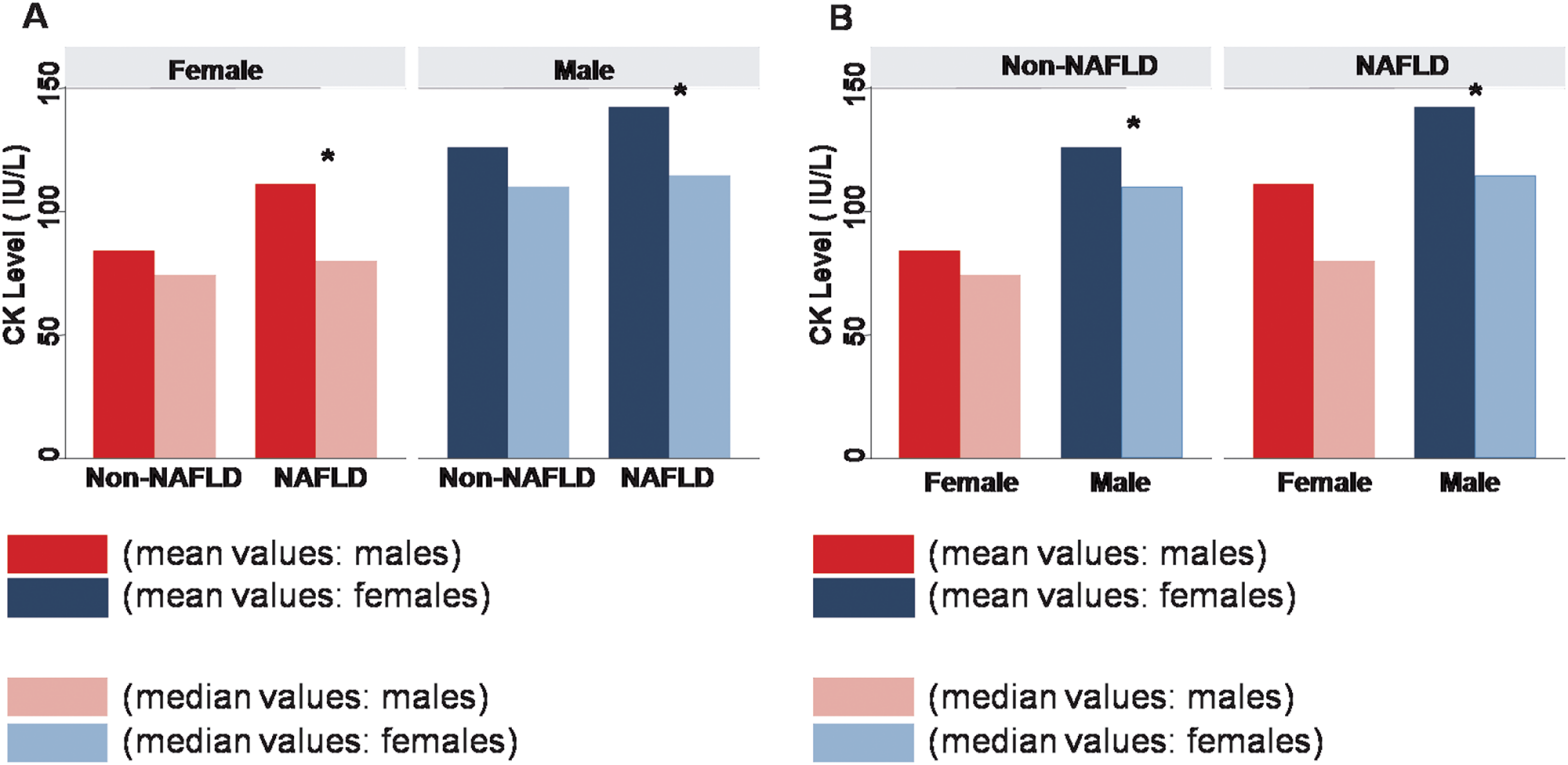
The existence of NAFLD appears to be associated with higher serum CK levels in both genders (left panel), with males showing larger CK levels than females with or without NAFLD (right panel). Data were expressed as either mean or median stratified by genders or presence of NAFLD. * denotes p <0.05 in comparisons between subjects with or without NAFLD, or comparisons between genders by T-test.

Multivariate associations between CK levels, blood pressure components, and various body adiposity measures and their potential gender interactions were also examined (Table 4 and S2 Table). To examine the potential gender-related modifications on CK activity, interaction analysis was conducted. Gender was shown to be a strong modifier of the relationship between circulating CK levels with age. CK levels showed a modest inverted relationship with age in men though increased with age in women (p for interaction: < .001) (Fig 1, Table 4 and S2 Table). Furthermore, the association between elevated CK levels and higher SBP was significant in men (all p < .05), but not in women (Fig 1, Table 4). Most associations between CK levels and PP (but not DBP) also demonstrated gender differences in multivariate models, with PP associated with CK levels in men rather than women (Fig 1, S2 Table). Finally, while elevated CK level was independently associated with larger BMI or fat mass in both genders, these associations were more pronounced in men than women (all interaction p < .05), with the exception of waist circumference (Table 4 and S2 Table). Of note, the presence of NAFLD was also significantly associated with elevated CK levels in men (NAFLD: mean 142.2 ± 6.6 vs Non-NAFLD: 125.6±1.6 IU/L, p < .001) and in women (NAFLD: mean 111 ± 17.5 vs Non-NAFLD: 84.2±1.1 IU/L, both p < .05) with no gender modification effects (Fig 3).

**Table 4:** Multivariate associations of CK with SBP and various adiposity measures with sex stratification.

Gender	Male		Female		P for sex interaction
Predictors	*Coef*.	*P value*	*Coef*.	*P value*	
**BMI (kg/m**^**2**^**)**^#^					
Age (per 10 years +)	-5.62	<0.001	2.84	0.01	†
SBP (per 10 mmHg +)	2.59	0.021	0.39	0.634	†
BMI (per unit +)	3.72	<0.001	1.19	<0.001	†
eGFR (per 10 unit +)	-3.77	<0.001	-3.47	<0.001	NS
**Waist (cm)**^#^					
Age (per 10 years +)	-7.04	<0.001	2.78	0.014	†
SBP (per 10 mmHg +)	2.6	0.02	0.54	0.504	†
Waist (per 10 unit +)	9.29	<0.001	3.86	0.002	NS
eGFR (per 10 unit +)	-3.82	<0.001	-3.42	<0.001	NS
**Fat Mass (kg)**^#^					
Age (per 10 years +)	-5.13	<0.001	3.27	0.003	†
SBP (per 10 mmHg +)	2.81	0.012	0.53	0.513	†
FM (per 5kg +)	5.8	<0.001	2.23	0.005	†
eGFR (per 10 unit +)	-3.68	<0.001	-3.5	<0.001	NS
**NAFLD**^#^					
Age (per 10 years +)	-8.31	<0.001	5.32	<0.001	†
SBP (per 10 mmHg +)	5.05	<0.001	0.57	0.59	†
NAFLD	10.85	0.039	25.6	<0.001	NS
eGFR (per 10 unit +)	-5.65	<0.001	-3.73	<0.001	NS

*Abbreviations as* Tables 1–3. *Note*: Regression coefficients (*β*) represent the change in mean difference in CK (in IU/L) per 1-SD difference in each continuous predictor variable. *Other abbreviations as* Table 1.

^†^ sex interactions p<0.05;

^#^ Further adjusted for current smoker, hypertension, hyperlipidemia treatment and diabetes history.

## Discussion

This study is the first of its kind to explore the clinical correlates of circulating CK levels in a large asymptomatic Asian cohort. As in cohorts of other ethnicities, we found a positive correlation between CK levels and blood pressure; however, after multivariate adjustment, this relationship remained significant in men, but not in women. Overall, higher circulating CK levels were related to larger anthropometric measures or greater adiposity indexes in terms of NAFLD and worsening renal function. In addition, the relationships between higher circulating CK level, higher blood pressure, and several anthropometric were stronger in men than women. Of note, gender was an important modifier of the relationship between CK levels and age, as well as between CK levels, and body anthropometric data, such as BMI or fat mass.

### Associations between circulating CK and blood pressure

The association between high CK activity and high blood pressure has been well-described in Western populations [2,3,5,13–14]. Potential mechanisms underlying this association include higher enzyme activity in resistance arteries^3^ fueling CK-related energy demanding ATPase in contractile myosin proteins (such as Na^1+^/K^1+^-ATPase or Ca^2+^-ATPase) [2,15,16], enhancing pressor effects in the presence of smooth muscle proliferation in hypertensive states [17,18]. In fact, variations in CK activity have been postulated to account for blood pressure differences between black and white populations, where higher CK levels in the former may explain their predisposition to hypertension [19].

### Associations between circulating CK and anthropometric or adiposity measures

Elevated serum CK activity has been suggested as indicative of coupled striated skeletal muscle mass and activity, specifically type II fibers which are characterized by high cytosolic CK activity [15]. Type II fibers utilize different metabolic and energy processing pathways compared with type I fibers. For example, type II fibers have lower mitochondrial content, lower expression of insulin-dependent transporter protein GLUT-4 [1, 3, 20–22], and lower utilization of fatty acid oxidation [23–25]. Therefore, high circulating CK levels, associated with a predominance of type II fibers, may indicate greater propensity to excessive lipid storage [26,27]. Consistent with these postulations, we demonstrated that higher circulating CK levels were associated with larger body size, higher body fat mass, and clinical evidence of various excessive adiposity measures (characterized by greater waist circumference and the presence of NAFLD) [28]. Though greater body mass was strongly associated with greater circulating CK level, we noticed no significant associations between type 2 DM prevalence and CK concentration, which is consistent to prior reports [29]. As CK is largely influenced by total body mass and tissue turnover, the presence of greater proportions of lean individuals with poorly controlled type 2 diabetes may explain the apparent indirect relationship between blood sugar and CK level. Furthermore, we also demonstrated that NAFLD, an alternative clinical marker of adiposity which plays a pivotal role in the regulation of carbohydrate and lipoprotein metabolism, was strongly associated with elevated CK levels [30]. CK elevation in patients with NAFLD may be due to similar mechanisms as those observed in patients with metabolic triglyceride overflow (i.e., a type II skeletal muscle fiber predominant individual) [31,32].

### Gender-differences in CK activity

Age- and gender-related differences in blood CK levels have been previously reported [19,33]. Sex hormones may partially explain the divergent CK relationships found in several clinical covariates between men and women. Estrogen in women regulates several biological pathways that determine fat distribution and body compositions, and may impact and attenuates glucose and fatty acid oxidative metabolism in skeletal muscle [34,35]. Indeed, we observed that male gender presents with higher CK levels that correlate with greater fat mass or BMI compared with female gender. It has also been proposed that alterations in hormonal status may directly affect CK levels and mediate associated changes via CK-independent pathways after menopause [36, 37]. Further, higher serum levels of testosterone in men is associated with increased skeletal muscle mass and have been associated with elevated CK levels and enhanced skeletal muscle energy metabolism in men compared with women of the same age [38]. Therefore, declining testosterone levels in elderly men may result in a contradictory reduction in CK levels compared with elderly women. Although statin usage has been demonstrated to be a major cause of elevated serum CK levels in clinical settings [39], in our current cohort, there was very low statin usage (<5%) and statins may not be a primary concern.

Taken together, our data supports previous reports and extends those data to a large asymptomatic Asian cohort. The current study provides the first evidence supporting the clinical relevance of CK levels as they correlate with blood pressure and body size (or various adiposity measures) in a large number of asymptomatic Asian adults. In addition, our findings may be helpful in guiding future research into the underlying etiology of elevated CK levels without known ongoing myocardial injury—an association which may be gender-related especially given the ongoing debate regarding the clinical value of assessing baseline circulating CK activity without any known ongoing myocardial damage. In particular, there is a paucity of data addressing the underlying clinical factors related to circulating CK levels in a large Asian population. We demonstrated that several key biological factors, including age and blood pressure, correlate highly with CK activity, although the interpretation of such associations can be different between genders. While women may have higher circulating CK levels with increasing age, men tend to show greater CK activity with higher blood pressure. Further, we consistently showed that larger body size or certain adiposity measures may be associated with higher CK activity, especially in men.

### Study limitations

Our study had several limitations including its cross-sectional nature which precludes conclusions regarding cause and effect. Measurement of sub-types of CK (e.g., CK-MB) may be revealing, but was not available in the current study. Furthermore, although a trend toward a subtle relationship between circulating CK levels and several biochemical data was noted, for example, uric acids and several lipid profiles, those associations failed to reach significance after accounting for other clinical covariates. Instead, age, renal function, body size, and adiposity measures remained strongly associated with circulating CK levels after confounder effects were considered. In addition, our study sample consisted of a uniformly asymptomatic adult population in whom use of medications such as statins was low (<5%). Further, the lack of longitudinal follow-up regarding these measures, especially the potential interactions between serum CK levels, body size, and various adiposity estimates in outcome-driven analysis, may still need future exploration.

## Conclusions

We provided data in a large asymptomatic Asian population which demonstrated that higher circulating CK levels may be age-related, and were associated with higher blood pressure, larger body anthropometric, and several adiposity measures. These relationships were stronger in men than women (with the exception of waist circumference or NAFLD). The underlying reasons for gender-related divergent relationships between CK levels and age, as well as between CK levels and certain adiposity measures deserve further study.

## Supporting information

S1 Table Multivariate associations of CK by DBP, PP and various anthropometric measurements. (DOC)

S2 Table Multivariate associations of CK with DBP, PP and various adiposity measures with sex stratification. (DOCX)

## References

[1] Dzeja PP, Terzic A. Phosphotransfer networks and cellular energetics. J Exp Biol. 2003;206: 2039–2047. 12756286 10.1242/jeb.00426

[2] Brewster LM, Mairuhu G, Bindraban NR, Koopmans RP, Clark JF, van Montfrans GA. Creatine kinase activity is associated with blood pressure. Circulation. 2006;114:2034–2039. doi: 10.1161/CIRCULATIONAHA.105.584490 17075013 10.1161/CIRCULATIONAHA.105.584490

[3] Brewster LM, Clark JF, van Montfrans GA. Is greater tissue activity of creatine kinase the genetic factor increasing hypertension risk in black people of sub-Saharan African descent? J Hypertens. 2000;18:1537–1544. 11081764 10.1097/00004872-200018110-00002

[4] Brewster LM, Coronel CM, Sluiter W, Clark JF, van Montfrans GA. Ethnic differences in tissue creatine kinase activity: an observational study. PLoS One. 2012;7: e32471 doi: 10.1371/journal.pone.0032471 22438879 10.1371/journal.pone.0032471 PMC3306319

[5] Hittel DS, Hathout Y, Hoffman EP, Houmard JA. Proteome analysis of skeletal muscle from obesity and morbidly obese women. Diabetes. 2005;54: 1283–1288. 15855311 10.2337/diabetes.54.5.1283

[6] Hickey MS, Carey JO, Azevedo JL, Houmard JA, Pories WJ, Israel RG, et al Skeletal muscle fiber composition is related to adiposity and in vitro glucose transport rate in humans. Am J Physiol. 1995; 268: E453–E457. 7900793 10.1152/ajpendo.1995.268.3.E453

[7] Hulver MW, Berggren JR, Cortright RN, Dudek RW, Thompson RP, Pories WJ, et al Skeletal muscle lipid metabolism with obesity. Am J Physiol Endocrinol Metab. 2003; 284: E741–E747. doi: 10.1152/ajpendo.00514.2002 12626325 10.1152/ajpendo.00514.2002

[8] He J, Watkins S, Kelley DE. Skeletal muscle lipid content and oxidative enzyme activity in relation to muscle fiber type in type 2 diabetes and obesity. Diabetes. 2001;50: 817–823. 11289047 10.2337/diabetes.50.4.817

[9] Kotani K, Tokunaga K, Fujioka S, Kobatake T, Keno Y, Yoshida S, et al Sexual dimorphism of age-related changes in whole-body fat distribution in the obese. Int J Obes Rela Metab Disord. 1994;18: 207–212. 8044194

[10] Parekh S, Anania FA. Abnormal lipid and glucose metabolism in obesity: implications for nonalcoholic fatty liver disease. Gastroenterology. 2007;132: 2191–2207. doi: 10.1053/j.gastro.2007.03.055 17498512 10.1053/j.gastro.2007.03.055

[11] Omagari K, Kadokawa Y, Masuda J et al Fatty liver in non-alcoholic non-overweight Japanese adults: incidence and clinical characteristics. J. Gastroenterol. Hepatol. 2002; 17: 1098–105 12201871 10.1046/j.1440-1746.2002.02846.x

[12] Chen CH, Huang MH, Yang JC et al Prevalence and risk factors of nonalcoholic fatty liver disease in an adult population of Taiwan: metabolic significance of nonalcoholic fatty liver disease in nonobese adults. J. Clin. Gastroenterol. 2006; 40: 745–52. 16940890 10.1097/00004836-200609000-00016

[13] Garcia W. Elevated creatine phosphokinase levels: association with large muscle mass. Another pitfall in evaluating clinical significance of total serum CPK activity. JAMA. 1974;228: 1395–1396. 4406615

[14] Johnsen SH, Lilleng H, Wilsgaard T, Bekkelund SI. Creatine kinase activity and blood pressure in a normal population: the Tromsø study. J Hypertens. 2011;29: 36–42. doi: 10.1097/HJH.0b013e32834068e0 21063205 10.1097/HJH.0b013e32834068e0

[15] Wallimann T, Dolder M, Schlattner U, Eder M, Hornemann T, O’Gorman E, et al Some new aspects of creatine kinase (CK): compartmentation, structure, function and regulation for cellular and mitochondrial bioenergetics and physiology. Biofactors. 1998;8: 229–234. 9914824 10.1002/biof.5520080310

[16] Clark JF. The creatine kinase system in smooth muscle. Mol Cell Biochem. 1994;133–134: 221–232. 7808455 10.1007/BF01267956

[17] Somjen D, Kohen F, Jaffe A, Amir-Zaltsman Y, Knoll E, Stern N. Effects of gonadal steroids and their antagonists on DNA synthesis in human vascular cells. Hypertension. 1998;32:39–45. 9674635 10.1161/01.hyp.32.1.39

[18] Hadjiisky P, Bourdillon MC, Grosgogeat Y. Enzyme histochemical expressions of smooth muscle cell modulation in arterial development, hypertension and remodeling. Cell Mol Biol. 1991;37: 531–540. 1934022

[19] Neal RC, Ferdinand KC, Ycas J, Miller E. Relationship of ethnic origin, gender, and age to blood creatine kinase levels. Am J Med. 2009;122: 73–78. doi: 10.1016/j.amjmed.2008.08.033 19114174 10.1016/j.amjmed.2008.08.033

[20] Sousa De, Veksler V, Bigard X, Mateo P, Ventura-Clapier R. Heart failure affects mitochondrial but not myofibrillar intrinsic properties of skeletal muscle. Circulation. 2000;102: 1847–1853. 11023942 10.1161/01.cir.102.15.1847

[21] Clark JF, Khuchua Z, Kuznetsov AV, Vassil'eva E, Boehm E, Radda GK, et al Actions of the creatine analogue beta guanidinopropionic acid on rat heart mitochondria. Biochem J. 1994;300: 211–216. 8198536 10.1042/bj3000211 PMC1138144

[22] Pandke KE, Mullen KL, Snook LA, Bonen A, Dyck DJ. Decreasing intramuscular phosphagen content simultaneously increases plasma membrane FAT/CD36 and GLUT4 transporter abundance. Am J Physiol Regul Integr Comp Physiol. 2008;295: R806–R813. doi: 10.1152/ajpregu.90540.2008 18650314 10.1152/ajpregu.90540.2008

[23] Sun G, Ukkola O, Rankinen T, Joanisse DR, Bouchard C. Skeletal muscle characteristics predict body fat gain in response to overfeeding in never-obese young men. Metabolism. 2002;51: 451–456. 11912552 10.1053/meta.2002.31324

[24] Lindena J, Diederichs F, Wittenberg H, Trautschold I. Kinetic of adjustment of enzyme catalytic concentrations in the extracellular space of the man, the dog and the rat. Approach to a quantitative diagnostic enzymology, V. Communication. J Clin Chem Clin Biochem. 1986;24: 61–71. 3517220 10.1515/cclm.1986.24.1.61

[25] Clarkson PM, Kroll W, Graves J, Record WA. The relationship of serum creatine kinase, fiber type, and isometric exercise. Int J Sports Med. 1982;3: 145–148. doi: 10.1055/s-2008-1026078 7129722 10.1055/s-2008-1026078

[26] Tanner CJ, Barakat HA, Dohm GL, Pories WJ, MacDonald KG, Cunningham PR, et al Muscle fiber type is associated with obesity and weight loss. Am J Physiol Endocrinol Metab. 2002;282: E1191–E1196. doi: 10.1152/ajpendo.00416.2001 12006347 10.1152/ajpendo.00416.2001

[27] Wade AJ, Marbut MM, Round JM. Muscle fibre type and aetiology of obesity. Lancet. 1990;335: 805–808. 1969558 10.1016/0140-6736(90)90933-v

[28] Despres JP, Lemieux I, Prud’homme D. Treatment of obesity: need to focus on high risk abdominally obese patients. BMJ. 2001;322: 716–720. 11264213 10.1136/bmj.322.7288.716 PMC1119905

[29] Brewster LM, Clark JF, van Montfrans GA. Is greater tissue activity of creatine kinase the genetic factor increasing hypertension risk in black people of sub-Saharan African descent? J Hypertens. 2000;18:1537–1544. 11081764 10.1097/00004872-200018110-00002

[30] Siegelman ES, Rosen MA. Imaging of hepatic steatosis. Semin Liver Dis. 2001;21: 71–80. 11296698 10.1055/s-2001-12930

[31] Executive summary of the Third Report of the National Cholesterol Education Program (NCEP) Expert Panel on Detection, Evaluation, and Treatment of High Blood Cholesterol in Adults (Adult Treatment Panel III). JAMA. 2001;285: 2486–2497. 11368702 10.1001/jama.285.19.2486

[32] Gogia S, Neuschwander-tetri BA. Unexplained CK elevations in patient with non-alcoholic steatohepatitis. Liver Int. 2006;26: 899–900. doi: 10.1111/j.1478-3231.2006.01299.x 16911474 10.1111/j.1478-3231.2006.01299.x

[33] Wong ET, Cobb C, Umehara MK, Wolff GA, Haywood LJ, Greenberg T, et al Heterogeneity of serum creatine kinase activity among racial and gender groups of the population. Am J Clin Pathol. 1983;79: 582–586. 6837521 10.1093/ajcp/79.5.582

[34] Bryzgalova G, Gao H, Ahren B, Zierath JR, Galuska D, Steiler TL, et al Evidence that oestrogen receptor-alpha plays an important role in the regulation of glucose homeostasis in mice: insulin sensitivity in the liver. Diabetologia. 2006;49: 588–597. doi: 10.1007/s00125-005-0105-3 16463047 10.1007/s00125-005-0105-3

[35] Ribas V, Nguyen MT, Henstridge DC, Nguyen AK, Beaven SW, Watt MJ, et al Impaired oxidative metabolism and inflammation are associated with insulin resistance in ER alpha-deficient mice. Am J Physiol Endocrinol Metab. 2010;298: E304–E319. doi: 10.1152/ajpendo.00504.2009 19920214 10.1152/ajpendo.00504.2009 PMC2822483

[36] Gutierrez-Grobe Y, Ponciano-Rodríguez G, Ramos MH, Uribe M, Méndez-Sánchez N. Prevalence of non alcoholic fatty liver disease in premenopausal, postmenopausal and polycystic ovary syndrome women. The role of estrogens. Ann Hepatol. 2010;9: 402–409. 21057159

[37] Shimizu I, Kohno N, Tamaki K, Shono M, Huang HW, He JH, et al Female hepatology: favorable role of estrogen in chronic liver disease with hepatitis B virus infection. World J Gastroenterol. 2007;13: 4295–4305. doi: 10.3748/wjg.v13.i32.4295 17708600 10.3748/wjg.v13.i32.4295 PMC4250853

[38] Ramamani A, Aruldhas MM, Govindarajulu P. Impact of testosterone and oestradiol on region specificity of skeletal muscle-ATP, creatine phosphokinase and myokinase in male and female Wistar rats. Acta Physiol Cand. 1999;166: 91–97. 10.1046/j.1365-201x.1999.00576.x 10383487

[39] Gogia S. Unexplained CK elevation in patients with nonalcoholic steatohepatitis. Liver Int. 2006;26: 899–890. 16911474 10.1111/j.1478-3231.2006.01299.x

