# Supplementary material for: Gender-differences in the associations between circulating creatine kinase, blood pressure, body mass and non-alcoholic fatty liver disease in asymptomatic asians
Source: PLoS One. 2017 Jun 30;12(6):e0179898. doi: 10.1371/journal.pone.0179898 (PMC5493338; doi:10.1371/journal.pone.0179898)
Supplement: S1 Table — (DOC) [file pone.0179898.s001.doc]

**S1 Table. Multivariate associations of CK with DBP, PP and various anthropometric measurements**

| **Predictors** | ***Coef.*** | ***95% CI*** | ***P value*** |
| --- | --- | --- | --- |
| **BMI (kg/m^2^)^＃^** |  |  |  |
| Age (per 10 years +) | -2.17 | -3.83 to -0.51 | 0.01 |
| Gender, Male | 35.5 | 31.8 to 39.2 | <0.001 |
| DBP (per 10 mmHg +) | 0.72 | -1.79 to 3.24 | 0.572 |
| BMI (per unit +) | 2.69 | 2.17 to 3.21 | <0.001 |
| eGFR (per 10 unit +) | -3.79 | -4.82 to -2.76 | <0.001 |
| **Waist (cm)^＃^** |  |  |  |
| Age (per 10 years +) | -2.68 | -4.35 to -1.0 | 0.002 |
| Gender, Male | 31.4 | 25.7 to 35.9 | <0.001 |
| DBP (per 10 mmHg +) | 0.76 | -1.24 to 2.74 | 0.458 |
| Waist (per 10 unit +) | 7.62 | 5.63 to 9.61 | <0.001 |
| eGFR (per 10 unit +) | -3.73 | -4.76 to -2.71 | <0.001 |
| **Fat Mass (kg)^＃^** |  |  |  |
| Age (per 10 years +)^※^ | -1.34 | -3.02 to 0.33 | 0.115 |
| Gender, Male | 39.9 | 36.2 to 43.6 | <0.001 |
| DBP (per 10 mmHg +) | 1.37 | -1.15 to 3.89 | 0.287 |
| FM (per 5kg +) | 4.67 | 3.30 to 6.04 | <0.001 |
| eGFR (per 10 unit +) | -3.73 | -4.78 to -2.68 | <0.001 |
| **NAFLD^＃^** |  |  |  |
| Age (per 10 years +) | -1.78 | -3.87 to -0.24 | 0.038 |
| Gender, Male | 37.6 | 33.0 to 42.2 | <0.001 |
| DBP (per 10 mmHg +) | 2.22 | -0.26 to 4.71 | 0.08 |
| NAFLD | 16.3 | 8.44 to 24.1 | <0.001 |
| eGFR (per 10 unit +) | -4.69 | -5.98 to -3.39 | <0.001 |
|  |  |  |  |
|  |  |  |  |
| **Predictors** | ***Coef.*** | ***95% CI*** | ***P value*** |
| **BMI (kg/m^2^)^＃^** |  |  |  |
| Age (per 10 years +) | -2.95 | -4.68 to -1.22 | 0.001 |
| Gender, Male | 35.5 | 31.8 to 39.2 | <0.001 |
| PP (per 10 mmHg +) | 3.7 | 1.50 to 5.82 | 0.001 |
| BMI (per unit +) | 2.69 | 2.19 to 3.20 | <0.001 |
| eGFR (per 10 unit +) | -3.82 | -4.85 to -2.79 | <0.001 |
| **Waist (cm)^＃^** |  |  |  |
| Age (per 10 years +) | -3.85 | -6.04 to -1.67 | 0.001 |
| Gender, Male | 31.1 | 26.1 to 36.2 | <0.001 |
| PP (per 10 mmHg +) | 3.46 | 1.30 to 5.61 | 0.002 |
| Waist (per 10 unit +) | 7.87 | 5.42 to 10.32 | <0.001 |
| eGFR (per 10 unit +) | -4.72 | -6.0 to 3.44 | <0.001 |
| **Fat Mass (kg)^＃^** |  |  |  |
| Age (per 10 years +) | -2.11 | -3.85 to -0.36 | 0.018 |
| Gender, Male | 40.2 | 36.7 to 43.8 | <0.001 |
| PP (per 10 mmHg +) | 2.55 | 0.81 to 4.29 | 0.004 |
| FM (per 5kg +) | 4.8 | 3.47 to 6.14 | <0.001 |
| eGFR (per 10 unit +) | -3.77 | -4.81 to -2.72 | <0.001 |
| **NAFLD^＃^** |  |  |  |
| Age (per 10 years +) | -2.76 | -4.94 to -0.57 | 0.013 |
| Gender, Male | 38.4 | 33.9 to 42.9 | <0.001 |
| PP (per 10 mmHg +) | 3.46 | 1.29 to 5.63 | 0.002 |
| NAFLD | 16.7 | 8.93 to 24.6 | <0.001 |
| eGFR (per 10 unit +) | -4.73 | -6.02 to -3.44 | <0.001 |

Abbreviations as Table 1-3.

*Note*: Regression coefficients (*β*) represent the change in mean difference in CK (in IU/L) per 1-SD difference in each continuous predictor variable.

*Other abbreviations as Table 1*.

**^＃^** Further adjusted for current smoker, hypertension, hyperlipidemia treatment and diabetes history.
