# Supplementary material for: Gender-differences in the associations between circulating creatine kinase, blood pressure, body mass and non-alcoholic fatty liver disease in asymptomatic asians
Source: PLoS One. 2017 Jun 30;12(6):e0179898. doi: 10.1371/journal.pone.0179898 (PMC5493338; doi:10.1371/journal.pone.0179898)
Supplement: S2 Table — (DOCX) [file pone.0179898.s002.docx]

**S2 Table. Multivariate associations of CK with DBP, PP and various adiposity measures with sex stratification**

| **Gender** | **Male** | | **Female** | | **P for sex interaction** |
| --- | --- | --- | --- | --- | --- |
| **Predictors** | ***Coef.*** | ***P value*** | ***Coef.*** | ***P value*** |  |
| **BMI (kg/m^2^)^＃^** |  |  |  |  |  |
| Age (per 10 years +) | -5.03 | <0.001 | 3.13 | 0.003 | † |
| DBP (per 10 mmHg +) | 0.07 | 0.963 | -0.55 | 0.647 | NS |
| BMI (per unit +) | 3.82 | <0.001 | 1.22 | <0.001 | † |
| eGFR (per 10 unit +) | -3.69 | <0.001 | -3.47 | <0.001 | NS |
| **Waist (cm)^＃^** |  |  |  |  |  |
| Age (per 10 years +) | -6.44 | <0.001 | 4.6 | 0.001 | † |
| DBP (per 10 mmHg +) | 0.8 | 0.61 | 1.29 | 0.411 | NS |
| Waist (per 10 unit +) | 9.52 | <0.001 | 4.01 | 0.015 | NS |
| eGFR (per 10 unit +) | -3.76 | <0.001 | -3.66 | <0.001 | NS |
| **Fat Mass (kg)^＃^** |  |  |  |  |  |
| Age (per 10 years +) | -4.46 | 0.001 | 3.61 | 0.001 | † |
| DBP (per 10 mmHg +) | 0.92 | 0.559 | -0.27 | 0.826 | NS |
| FM (per 5kg +) | 5.92 | <0.001 | 2.27 | 0.005 | † |
| eGFR (per 10 unit +) | -3.62 | <0.001 | -3.49 | <0.001 | NS |
| **NAFLD^＃^** |  |  |  |  |  |
| Age (per 10 years +) | -7.14 | <0.001 | 5.53 | <0.001 | † |
| DBP (per 10 mmHg +) | 2.01 | 0.30 | 1.21 | 0.437 | NS |
| NAFLD | 11.69 | 0.027 | 25.6 | 0.45 | † |
| eGFR (per 10 unit +) | -5.53 | <0.001 | -3.73 | <0.001 | NS |
|  |  | | | |  |
| **Gender** | **Male** | | **Female** | | **P for sex interaction** |
| **Predictors** | ***Coef.*** | ***P value*** | ***Coef.*** | ***P value*** |  |
| **BMI (kg/m^2^)^＃^** |  |  |  |  |  |
| Age (per 10 years +) | -5.91 | <0.001 | 2.63 | 0.018 | † |
| PP (per 10 mmHg +) | 3.6 | 0.007 | 1.04 | 0.335 | † |
| BMI (per unit +) | 3.85 | <0.001 | 1.21 | 0.002 | † |
| eGFR (per 10 unit +) | -3.79 | <0.001 | -3.48 | <0.001 | NS |
| **Waist (cm)^＃^** |  |  |  |  |  |
| Age (per 10 years +) | -7.25 | <0.001 | 2.65 | 0.02 | † |
| PP (per 10 mmHg +) | 3.1 | 0.019 | 1.01 | 0.353 | † |
| Waist (per 10 unit +) | 9.69 | <0.001 | 3.95 | 0.001 | NS |
| eGFR (per 10 unit +) | -3.84 | <0.001 | -3.43 | <0.001 | NS |
| **Fat Mass (kg)^＃^** |  |  |  |  |  |
| Age (per 10 years +) | -5.26 | <0.001 | 3.12 | 0.005 | † |
| PP (per 10 mmHg +) | 3.32 | 0.012 | 1.07 | 0.322 | † |
| FM (per 5kg +) | 6.14 | <0.001 | 2.31 | 0.003 | † |
| eGFR (per 10 unit +) | -5.69 | <0.001 | -3.51 | <0.001 | NS |
| **NAFLD^＃^** |  |  |  |  |  |
| Age (per 10 years +) | -8.60 | <0.001 | 5.58 | <0.001 | † |
| PP (per 10 mmHg +) | 5.71 | 0.001 | -0.07 | 0.962 | † |
| NAFLD | 11.9 | 0.023 | 26.3 | <0.001 | NS |
| eGFR (per 10 unit +) | -5.66 | <0.001 | -3.73 | <0.001 | NS |

*Abbreviations:*

eGFR= estimated glomerular filtration rate; CK=creatine kinase; DBP=diastolic blood pressure; PP=pulse pressure; Coef=coefficient; CI=confidence interval.

*Note*: Regression coefficients (*β*) represent the change in mean difference in CK (in IU/L) per 1-SD difference in each continuous predictor variable.

*Other abbreviations as Table 1*.

† sex interactions p<0.05;

**^＃^** Further adjusted for current smoker, hypertension, hyperlipidemia treatment and diabetes history.
